# The role of vitamin D deficiency in fetal growth restriction: a systematic review

**DOI:** 10.3389/fmed.2025.1725177

**Published:** 2026-01-13

**Authors:** Leony Octavia, Putri Nabila, Dwi Andhika Panjarwanto, Putri Lenggo Geany, Yosua Darmadi Kosen, Aldo Aulia Rahman, Vallexa Septina Yora, R. Mohamad Javier, Mohammad Haekal, Lucky Sutanto, Arthur Peter Tandayu, Srigita Varsha, Muhamad Dwi Eka Putra, Angela Anjelina Cita, Brilliant Sofia Maharani, Muhammad Falah Ghani Nuruddin, Savira Salsabila, Dwi Fitri Handayani Friska

**Affiliations:** 1 Faculty of Medicine, Hasanuddin University, Makassar, Indonesia; 2 Faculty of Medicine, Pelita Harapan University, Tangerang, Indonesia; 3 Faculty of Medicine, Brawijaya University, Malang, Indonesia; 4 Faculty of Medicine, Diponegoro University, Semarang, Indonesia; 5 Faculty of Medicine, Maranatha Christian University, Bandung, Indonesia; 6 Faculty of Medicine, Sriwijaya University, Palembang, Indonesia; 7 Department of Obstetrics and Gynecology, Fatmawati Hospital, Jakarta, Indonesia; 8 Faculty of Medicine, Muhammadiyah Malang University, Malang, Indonesia; 9 Division of Fertility, Endocrinology, and Reproductive Medicine, Department of Obstetrics and Gynecology, National Maternal and Child Health Center RSAB Harapan Kita, Jakarta, Indonesia; 10 Department of Obstetrics and Gynecology, Kartika Husada Tanjungpura Hospital, Tanjungpura, Indonesia; 11 Faculty of Medicine, Padjadjaran University, Bandung, Indonesia; 12 Faculty of Medicine, University of Jember, Jember, Indonesia; 13 Faculty of Medicine, Duta Wacana Christian University, Yogyakarta, Indonesia; 14 Faculty of Medicine, Sultan Agung University, Semarang, Indonesia; 15 Faculty of Medicine, Universitas Islam Indonesia, Yogyakarta, Indonesia; 16 Faculty of Medicine, Riau University, Pekanbaru, Indonesia

**Keywords:** birthweight, fetal growth restriction, placental angiogenesis, pregnancy outcomes, preterm birth, small-for-gestational-age, vitamin D deficiency

## Abstract

**Background:**

Fetal Growth Restriction (FGR) and Small-for-Gestational-Age (SGA) are major contributors to perinatal morbidity and mortality. Maternal vitamin D deficiency has been proposed to impair placental development and fetal growth through mechanisms involving angiogenesis, immune regulation, and oxidative stress. Increasing evidence suggests that maternal 25-hydroxyvitamin D [25(OH)D] status may play a significant role in the pathogenesis of impaired fetal growth.

**Objective:**

To synthesize evidence on the association between maternal 25-hydroxyvitamin D [25(OH)D] concentrations and the risk of FGR and SGA, and to evaluate the impact of vitamin D supplementation on fetal growth outcomes.

**Methods:**

A systematic search of PubMed, Cochrane Library, Springer, ScienceDirect, and DOAJ was performed following PRISMA 2020 guidelines. Observational studies and randomized controlled trials examining maternal vitamin D status and fetal growth outcomes were included.

**Results:**

The 48 included studies showed consistent evidence linking low maternal 25(OH)D concentrations with increased risk of FGR and SGA, with several analyses demonstrating dose–response patterns at lower vitamin D thresholds. Associations with preterm birth were directionally similar but less consistent. Findings from intervention trials assessing vitamin D supplementation were heterogeneous, influenced by variations in dosage, timing of initiation, baseline vitamin D status, adherence, and co-nutrient exposures. Experimental and mechanistic studies further supported biological plausibility, demonstrating vitamin D–mediated effects on placental angiogenesis, immune modulation, endocrine signaling, and oxidative stress pathways.

**Conclusion:**

Maternal vitamin D deficiency is consistently associated with impaired fetal growth, although findings from supplementation trials remain variable. Standardized dosing strategies, harmonized diagnostic cut-offs, and better-controlled interventions are needed to clarify the optimal role of vitamin D in preventing FGR and SGA.

**Systematic review registration:**

https://www.crd.york.ac.uk/prospero/, identifier CRD42024622395.

## 1 Introduction

Fetal growth restriction (FGR) occurs when a fetus fails to reach its biologically determined growth potential. It affects approximately 5%–10% of pregnancies and is a leading cause of perinatal mortality, neonatal morbidity, and long-term neuro-cardiometabolic complications (1–4). Early-onset FGR (<32 weeks) carries the poorest prognosis, including intrauterine fetal demise and long-term neurodevelopmental sequelae; current management is largely expectant, emphasizing surveillance and timing of delivery rather than disease-modifying therapy (4, 5).

Vitamin D is a fat-soluble steroid found in two main forms: vitamin D3 (cholecalciferol), synthesized in the skin after ultraviolet-B exposure and found in animal-derived foods, and vitamin D2 (ergocalciferol), which comes from plant and fungal sources. Both forms are hydroxylated in the liver to 25-hydroxyvitamin D [25(OH)D], the primary circulating biomarker of vitamin D status and further converted in the kidneys and placenta to the active 1,25-dihydroxyvitamin D [1,25(OH)_2_D] (6–9). During pregnancy, maternal vitamin D is the sole source for the fetus and contributes to skeletal development, placental function, and immune modulation; maternal 1,25(OH)_2_D rises progressively across gestation and peaks at delivery, predominantly driven by substrate availability [25(OH)D] rather than classical calcium homeostasis (6–9). Although 600 IU/day is commonly recommended, some populations may need higher doses (up to 4,000 IU/day) to improve maternal and neonatal outcomes (10).

Vitamin D deficiency during pregnancy is widespread globally and influenced by factors such as ethnicity, latitude, seasonality, obesity, diet, and limited sun exposure. Severe deficiency has been associated with preeclampsia, gestational diabetes, preterm birth, and impaired fetal growth (11–14). FGR itself defined as failure to achieve genetic growth potential remains a leading cause of perinatal morbidity and mortality; early-onset disease is particularly devastating and, at present, no therapy directly targets its pathophysiology beyond close monitoring and timely delivery (4, 5).

Biological plausibility for a vitamin D–FGR link is strong. Vitamin D promotes extravillous trophoblast invasion and spiral-artery remodeling, enhances placental angiogenesis, modulates maternal–fetal immune tolerance, and tempers oxidative and inflammatory signaling–processes fundamental to normal placentation and nutrient transfer (15–20). Experimental and translational data indicate that deficiency disrupts these pathways, contributing to placental insufficiency and suboptimal fetal growth (15–20).

### 1.1 PRISMA

Epidemiologic studies consistently associate lower maternal 25(OH)D with reduced birthweight, FGR, and–where assessed–SGA, with dose–response signals in several adjusted analyses (11–14, 21). Meta-analyses suggest prenatal vitamin D supplementation may improve birthweight and reduce low birthweight or SGA risk in some contexts. Yet, trial findings remain mixed owing to heterogeneity in dose, timing of initiation, baseline status, adherence, assays/cut-offs, and co-nutrient exposure (22–36).

Therefore, this study aims to systematically review current evidence on whether maternal vitamin D deficiency increases the risk of fetal growth restriction and related perinatal outcomes, and whether vitamin D supplementation may improve fetal growth parameters.

## 2 Materials and methods

### 2.1 Study design and protocol

**FIGURE 1:**
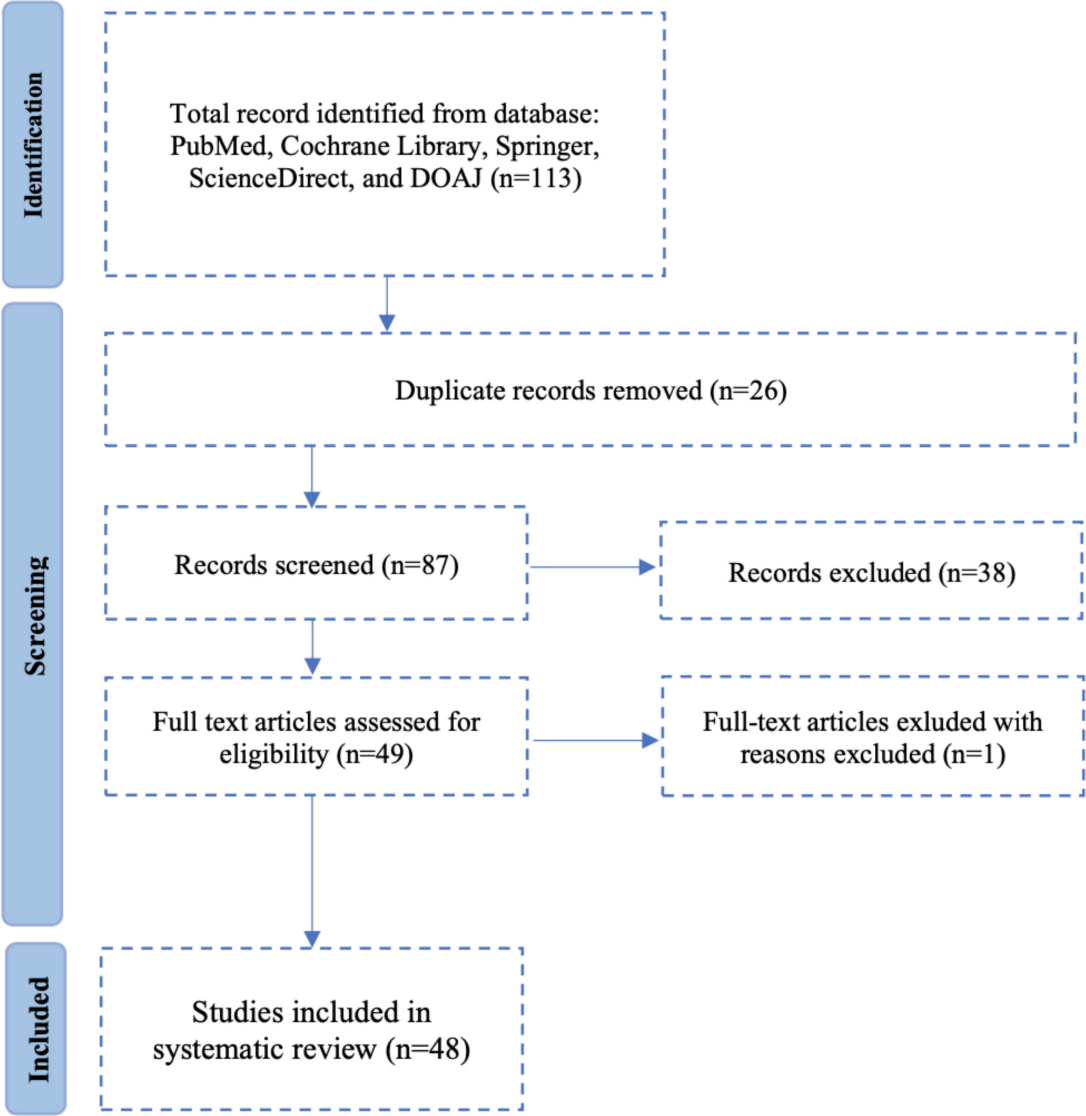
Systematic review diagram based on PRISMA.

This systematic review adhered to the PRISMA 2020 guidelines, and the study selection process is illustrated in Figure 1. The research protocol, based on the PICOS framework, was registered in PROSPERO (CRD42024622395). A comprehensive literature search was performed across PubMed, Cochrane Library, Scopus, ScienceDirect, Springer Nature, and DOAJ for articles published from January 2014 to December 2024. Only peer-reviewed studies in English or Indonesian were included. Two reviewers independently screened, selected, and extracted data; discrepancies were resolved by consensus or a third reviewer. Risk of bias was assessed using the Cochrane RoB 2.0 tool for RCTs and Newcastle-Ottawa Scale (NOS) for observational studies. Data synthesis was primarily narrative, with meta-analysis planned when sufficient homogeneous data permitted quantitative pooling.

### 2.2 Eligibility criteria (PICOS)

Population (P): Pregnant women with measured serum 25(OH)D.

Intervention (I): Maternal vitamin D deficiency or vitamin D supplementation.

Comparator (C): Adequate vitamin D status or placebo/no supplementation.

Outcomes (O): FGR, SGA, low birth weight (primary outcomes); preterm birth, perinatal mortality, neonatal morbidity (secondary outcomes).

Study design (S): RCTs, cohort, case-control, cross-sectional studies, systematic reviews/meta-analyses.

Inclusion required availability of fetal growth outcomes and maternal vitamin D measurement. Exclusion applied to narrative reviews, commentaries, studies without vitamin D data or fetal growth outcomes, abstract-only publications, duplicates, or those with inadequate outcome reporting or high risk of bias.

### 2.3 Search strategy

A systematic and comprehensive literature search was performed using six major electronic databases: PubMed, Cochrane Library, Scopus, ScienceDirect, Springer Nature, and DOAJ. The search covered peer-reviewed studies published between January 2014 and December 2024.

Search Terms and Boolean Operators

The following keywords and Boolean operators were used to refine the search:

- (“Vitamin D”) AND (“Vitamin D Deficiency”) AND (“Fetal Growth Restriction”)
- (“Maternal Vitamin D”) AND (“25-hydroxyvitamin D”) AND (“Pregnancy Outcomes”)
- (“Vitamin D Supplementation”) AND (“Birth Weight”)

The search was limited to studies published in English or Indonesian. Manual searches were also conducted in the references of relevant systematic reviews and meta-analyses to ensure comprehensive coverage.

### 2.4 Data extraction

Following the study selection process, data extraction was carried out independently by two reviewers to ensure accuracy and consistency. Extracted information included study characteristics such as author names, publication year, country, study design, sample size, and population demographics. Additionally, maternal vitamin D exposure was assessed by measuring serum 25(OH)D levels, which were categorized according to established deficiency thresholds. Outcome measures included FGR incidence, birth weight, SGA prevalence, and preterm birth rates. Adjustments for potential confounders, including maternal body mass index (BMI), ethnicity, and socioeconomic status, were also recorded.

To assess methodological quality, observational studies were evaluated using the Newcastle-Ottawa Scale (NOS), which assigns scores based on study selection, comparability, and outcome assessment. For RCTs, the Cochrane Risk of Bias Tool was applied to examine factors such as random sequence generation, allocation concealment, blinding, incomplete outcome data, and selective reporting. Any discrepancies in quality assessment between the two reviewers were resolved through discussion or by consulting a third reviewer.

## 3 Results

### 3.1 Distribution of papers

This systematic review involved a detailed examination of 48 studies exploring the link between vitamin D levels in patients with FGR. The studies were selected based on their relevance to the topic and their contribution to understanding the potential impact of baseline vitamin D levels on FGR incidences.

The distribution of the studies is categorized based on the type of study design, the population studied, and the primary outcomes measured. Tables 1–5 provides an overview of the distribution of the selected papers.

**TABLE 1:** Distribution of selected studies.

Study design	Number of studies	Key findings
Retrospective analysis	16	Analyzed historical cohort data to assess associations between maternal vitamin D levels and fetal growth outcomes, highlighting the impact of vitamin D deficiency on birth weight, gestational length, and pregnancy complications.
Randomized controlled trials	4	Evaluated the effects of prenatal vitamin D supplementation on maternal and fetal outcomes, with mixed results regarding its role in mitigating FGR. Some trials reported improvements in birth weight and fetal development, while others showed no significant impact.
Systematic reviews and meta-analyses	10	Synthesized global evidence from randomized controlled trials (RCTs) and observational studies, confirming a strong association between maternal vitamin D deficiency and FGR, but emphasizing the need for high-quality trials to establish causality.
Cross-sectional studies	5	Assessed maternal and neonatal vitamin D levels at a single time point, revealing significant correlations with FGR, but unable to determine causality due to study design limitations.
Case control	4	Identified strong associations between maternal vitamin D deficiency and FGR, with evidence suggesting genetic variations (VDR polymorphisms) may influence fetal growth outcomes.
Experimental studies	9	Investigated the biological mechanisms of vitamin D in FGR through animal models, *in vitro* studies, and clinical experiments, highlighting its role in placental function, oxidative stress reduction, and fetal development

**TABLE 2:** Distribution of reviewed papers by year.

Year	Number of papers
2014	2
2015	3
2016	4
2017	5
2018	5
2019	6
2020	6
2021	7
2022	4
2023	3
2024	3

**TABLE 3:** Distribution of studies by country.

Country	Number of studies	Percentage of total studies
United States	9	17.3%
China	7	13.5%
India	6	11.5%
United Kingdom	4	7.7%
Australia	3	5.8%
Brazil	3	3.8%
Egypt	2	3.8%
Iran	2	3.8%
Indonesia	1	1.9%
South Africa	1	1.9%
Germany	2	3.8%
Japan	1	1.9%
South Korea	1	1.9%
Canada	3	5.8%
Italy	2	3.8%
Spain	1	1.9%
France	1	1.9%
Mexico	1	1.9%
Turkey	1	1.9%
Pakistan	1	1.9%

**TABLE 4:** Summary of key studies on vitamin D and outcomes in FGR.

Study	Population	Key findings
Ding et al. (3)	Pregnant rats	Vitamin D3 supplementation mitigated oxidative stress and inflammation caused by PM2.5 exposure, improving fetal outcomes.
Xie et al. (19)	Pregnant mice	Vitamin D3 restored fetal weight and improved antioxidant enzyme activity, reducing fetal oxidative stress.
Ma et al. (23)	Pregnant rats	High-dose cholecalciferol reduced fetal weight and placental size, affecting placental function negatively.
Chen et al. (14)	Pregnant women	Vitamin D deficiency significantly increased the risk of spontaneous abortion and small-for-gestational-age births.

**TABLE 5:** Summary of key insights: relationship between vitamin D and FGR.

Study	Aspect	Vitamin D influence
Ding et al. (3)	Placental function	Vitamin D3 supplementation mitigated oxidative stress and improved fetal weight in cooking oil fume particulate matter (COF-PM2.5) exposed pregnancies.
Xie et al. (19)	Oxidative stress	Maternal Vitamin D3 supplementation in oxidized-oil diet reduced fetal oxidative stress and restored fetal weight.
Ma et al. (23)	Placental development	High-dose cholecalciferol inhibited trophoblast proliferation, reducing placental size and fetal growth.
Wang et al. (37)	Fetal lung development	Gestational Vitamin D deficiency impaired fetal lung growth by suppressing type II pneumocyte differentiation.
Tiwari et al. (38)	Genetic factors	VDR gene variants linked to increased risk of preterm birth and low birth weight.
Malm et al. (43)	Preeclampsia risk	Lower maternal Vitamin D levels in early pregnancy increased preeclampsia risk.

This table provides a structured summary of key findings from multiple studies, emphasizing the diverse pathways through which Vitamin D influences FGR. The evidence underscores the importance of maternal Vitamin D sufficiency in optimizing pregnancy outcomes by modulating placental function, reducing oxidative stress, supporting fetal lung development, and influencing genetic and hormonal pathways. Future research should focus on refining supplementation strategies and understanding long-term neonatal outcomes.

### 3.2 Key findings

The findings across different study designs underline the complex role of Vitamin D in fetal development and growth restriction:

1. Retrospective Analyses:

- Consistently linked low maternal Vitamin D levels to increased risk of FGR, spontaneous abortion, and SGA infants.
- Identified seasonal variations and sociodemographic factors influencing Vitamin D deficiency.
- Found associations between maternal 25(OH)D levels and neonatal birth weight, head circumference, and preterm birth.

2. Randomized Controlled Trials:

- Some trials demonstrated that Vitamin D supplementation improved pregnancy outcomes, including higher birth weights and reduced risk of FGR.
- Other studies reported inconsistent results, emphasizing the need for standardized supplementation protocols.

3. Systematic Reviews and Meta-Analyses:

- Reported significant associations between maternal Vitamin D levels and fetal growth restriction.
- Found variations in Vitamin D status based on maternal BMI, ethnicity, and nutritional intake.

4. Cross-Sectional Studies:

Reported significant associations between maternal Vitamin D levels and pregnancy complications such as preeclampsia and fetal growth restriction.
Found variations in Vitamin D status based on maternal BMI, ethnicity, and nutritional intake.

5. Case Control Studies:

Identified specific Vitamin D receptor (VDR) polymorphisms associated with fetal growth restriction and low birth weight.
Found that maternal Vitamin D deficiency was significantly associated with impaired fetal growth trajectories.

6. Experimental Studies:

Animal and *in vitro* studies demonstrated that Vitamin D deficiency disrupted placental function, increased oxidative stress, and impaired fetal organ development.
Vitamin D supplementation was found to mitigate growth restriction by enhancing placental vascularization, immune regulation, and nutrient transport.

### 3.3 Target of paper

This systematic review aims to evaluate the role of maternal Vitamin D deficiency in FGR by examining its impact on fetal development and underlying biological mechanisms. Specifically, this review seeks to:

- Assess the relationship between maternal Vitamin D levels and FGR risk, considering population-based and clinical studies.
- Investigate biological mechanisms, including placental function, oxidative stress pathways, and genetic influences that contribute to FGR.
- Discuss clinical implications, including potential screening strategies and Vitamin D supplementation approaches to mitigate FGR risk.

While the primary focus is on FGR, this review also considers how maternal Vitamin D status interacts with pregnancy complications such as placental insufficiency and fetal malnutrition, which are key contributors to growth restriction. By integrating findings from observational studies, randomized controlled trials, and systematic reviews, this paper aims to provide evidence-based insights and future research directions for optimizing maternal and fetal health.

### 3.4 Summary of findings

The findings from the 48 studies reviewed are summarized as follows:

#### Impact of Vitamin D on FGR:

- Ding et al. (3) demonstrated that Vitamin D3 supplementation protects against intrauterine growth restriction (IUGR) induced by cooking oil fume-derived particulate matter ≤ 2.5 μm (PM2.5) exposure by mitigating oxidative stress and inflammation.
- Xie et al. (19) found that maternal Vitamin D3 supplementation in an oxidized-oil diet protects fetal development and reduces oxidative stress in the placenta and fetus.

#### Effectiveness of Supplementation:

- Ma et al. (23) reported that high-dose cholecalciferol supplementation throughout pregnancy inhibited placental proliferation and contributed to fetal growth restriction.
- Chen et al. (14) provided strong evidence linking vitamin D deficiency to spontaneous abortion and SGA infants.

#### Biological Mechanisms:

- Wang et al. (37) found that gestational vitamin D deficiency impairs fetal lung development by suppressing type II pneumocyte differentiation.
- Tiwari et al. (38) explored maternal Vitamin D receptor (VDR) gene variants and their influence on preterm birth and fetal development.
- Malm et al. (39) highlighted the role of maternal serum vitamin D levels in early pregnancy and their association with preeclampsia risk.

**FIGURE 2:**
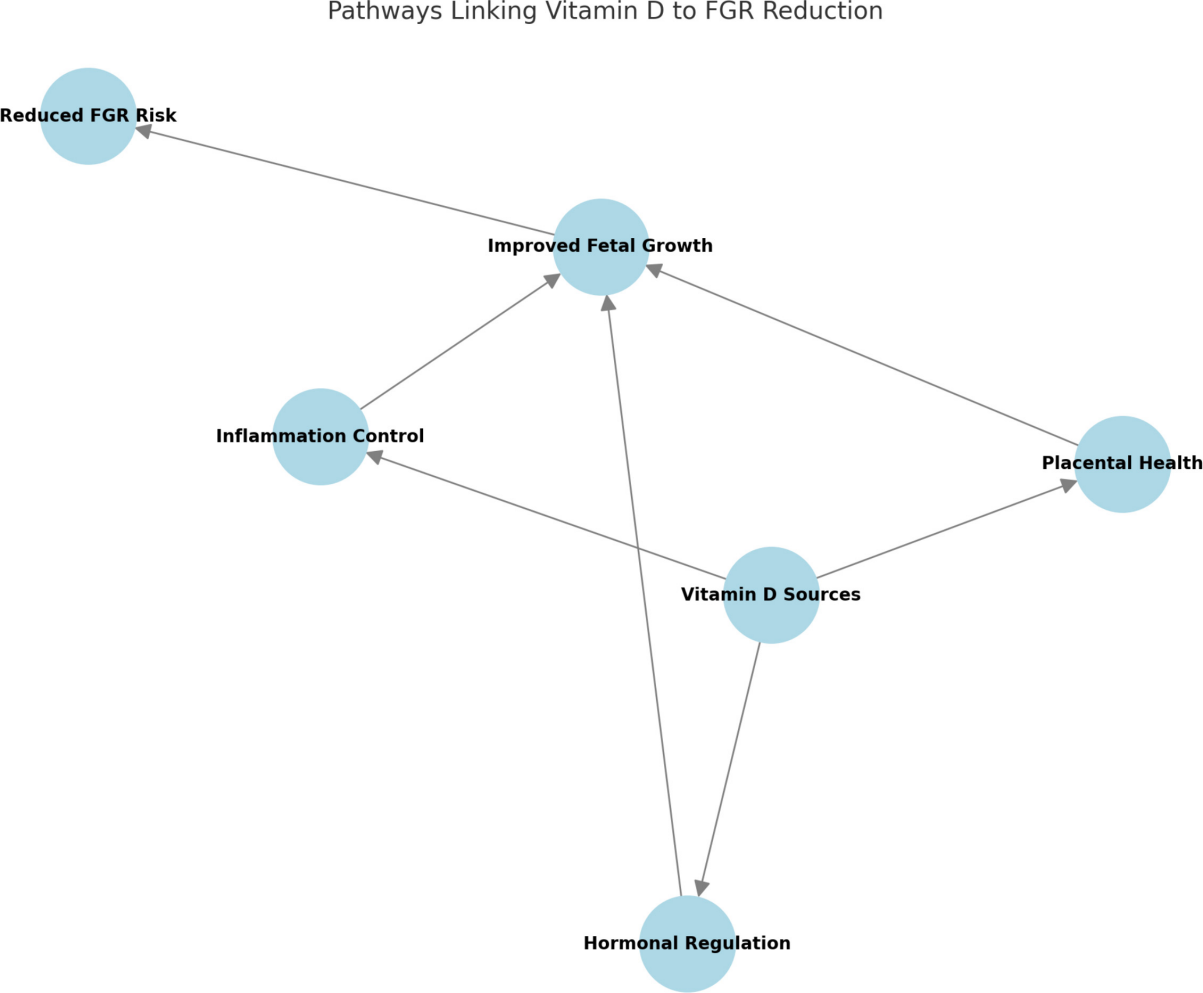
Pathways linking vitamin D to FGR reduction. This diagram illustrates the primary pathways through which Vitamin D contributes to the reduction of FGR risk. These pathways include placental health, hormonal regulation, and inflammation control, all of which support improved fetal growth.

The proposed biological mechanisms linking maternal vitamin D deficiency to fetal growth restriction, including placental angiogenesis, immune modulation, and oxidative stress pathways, are summarized in Figure 2.

#### Direct Impact on Placental Function:

- Studies indicate that Vitamin D deficiency impairs placental vascularization and nutrient transfer, leading to restricted fetal growth.
- Zhou et al. (40) found that Vitamin D supplementation improved placental blood flow, reducing FGR rates.

#### Role in inflammation and oxidative stress:

- Low Vitamin D levels are associated with increased pro-inflammatory cytokines (e.g., IL-6, TNF-alpha) and oxidative stress, which are linked to adverse pregnancy outcomes.
- Nguyen et al. (41) demonstrated that Vitamin D supplementation reduced inflammation markers, enhancing fetal development.

#### Hormonal Modulation:

- Vitamin D influences the production of insulin-like growth factor (IGF) and parathyroid hormone, both crucial for fetal growth.
- Studies by Li et al. (42) highlighted that adequate Vitamin D levels improve maternal metabolic profiles, indirectly supporting fetal development.

#### Clinical Implications:

The results emphasize the need for proactive management of Vitamin D levels during pregnancy, particularly in women at high risk for FGR. Clinical recommendations include:

- Regular screening for Vitamin D deficiency in prenatal care.
- Evidence-based supplementation to mitigate the risk of FGR and other adverse outcomes.

### 3.5 Analysis

The systematic review revealed several critical insights into the relationship between Vitamin D and FGR:

#### Consistency Across Studies:

- Retrospective and observational studies consistently report an inverse relationship between maternal Vitamin D levels and FGR risk.
- Systematic reviews and meta-analyses strengthen the evidence by synthesizing data from multiple populations and study designs.

#### Variability in Supplementation Outcomes:

- Randomized trials showed mixed results for Vitamin D supplementation. While most studies reported positive effects on birth weight and placental function, a few found no significant differences, likely due to:
  ∘    Variability in supplementation doses and protocols.
  ∘    Differences in baseline Vitamin D levels among study populations.

#### Regional and Demographic Factors:

- Studies from developing countries report higher prevalence rates of Vitamin D deficiency due to limited dietary intake and sun exposure, exacerbating FGR risks.
- Populations in temperate regions often face seasonal variations in Vitamin D levels, impacting pregnancy outcomes.

#### Gaps in Research:

- Longitudinal studies tracking the effects of maternal Vitamin D from early pregnancy to neonatal outcomes are scarce.
- Few studies focus on Vitamin D interactions with other micronutrients, such as calcium and magnesium, which may influence fetal growth.

Here, the review highlights findings from key studies that explore the relationship between Vitamin D levels and FGR outcomes:

These findings underscore the importance of maintaining optimal Vitamin D levels for mitigating risks associated with FGR.

### 3.6 Distribution of papers based on years

Publication trends from 2014 to 2024 indicate a steady increase in research exploring the role of Vitamin D in fetal growth restriction (FGR). The number of publications peaked between 2019 and 2024, highlighting the growing recognition of Vitamin D deficiency as a significant factor in maternal and fetal health. This trend reflects increased awareness of the need for routine Vitamin D assessment during pregnancy and its potential implications for fetal development.

This trend highlights the expanding understanding of Vitamin D’s role in maternal and fetal outcomes, emphasizing its significance in fetal development, placental function, and pregnancy complications. The increasing number of studies in recent years reflects the growing clinical and scientific interest in optimizing maternal Vitamin D levels to prevent FGR.

The systematic review analyzed studies from both developed and developing countries. The geographic distribution is summarized below:

The majority of studies were conducted in developed countries, reflecting a concentration of research resources and advanced maternal-fetal health research infrastructure. However, contributions from developing countries such as China, India, and Brazil underscore the global relevance of addressing Vitamin D deficiency during pregnancy. These findings highlight the need for further investigations in low-resource settings where maternal malnutrition and limited access to prenatal care may exacerbate the risk of FGR.

### 3.7 Visual data

Biological and Pathophysiological Insights

1. Placental Mechanisms: Vitamin D contributes to improved placental blood flow and nutrient exchange, which are critical for normal fetal growth. Studies suggest that Vitamin D regulates genes associated with trophoblast differentiation and angiogenesis, supporting a healthier placental environment.
2. Hormonal and Metabolic Influence: Key studies linked Vitamin D to improved insulin sensitivity, which supports better glucose transfer to the fetus. Observations included a positive effect on the production of growth-promoting hormones.
3. Anti-Inflammatory and Antioxidant Effects: Vitamin D reduces pro-inflammatory cytokines, such as IL-6 and TNF-alpha, which are often elevated in FGR cases. It also mitigates oxidative stress, protecting the placenta and fetal tissues from damage.

Recommendations for Clinical Practice

The systematic review highlights actionable recommendations for healthcare providers:

1. Routine Screening: Implement regular Vitamin D testing during prenatal visits, especially for high-risk populations prone to FGR.
2. Supplementation Guidelines: Develop standardized supplementation protocols tailored to individual Vitamin D status. For deficient cases, high-dose Vitamin D supplementation under clinical supervision is recommended.
3. Integration with Maternal Nutrition: Encourage balanced diets rich in Vitamin D (e.g., fortified foods, fatty fish). Highlight the role of sunlight exposure in improving Vitamin D synthesis naturally.

Research Gaps and Future Directions

Despite significant progress, the review identifies several gaps in the current research:

1. Inconsistencies in Supplementation Protocols: Varying doses and durations across studies make it difficult to establish universal recommendations.
2. Limited Longitudinal Data: A need for long-term studies that track Vitamin D effects from pregnancy to childhood.
3. Geographic and Socioeconomic Disparities: Most studies are concentrated in developed countries; further research is required in low-resource settings.

## 4 Discussion

This review indicates that lower maternal 25-hydroxyvitamin D [25(OH)D] is consistently associated with impaired fetal growth, primarily FGR and, where assessed, SGA, with several adjusted dose–response signals across diverse populations and study designs (1–5, 11–14, 21–29, 43–51). Findings for preterm birth were directionally similar but less uniform, reflecting clinical and methodological heterogeneity (30, 31, 43, 45–47). Randomized trials showed varied effects on birthweight and placental indices. These differences may be due to variations in dosage, timing of initiation, baseline vitamin D levels, adherence, assay methods, and co-nutrient exposure (4–6, 10–12, 25, 32, 37, 52–57). Taken together, maternal vitamin D deficiency emerges as a risk marker for impaired fetal growth rather than a definitively causal factor in unselected populations.

Biological plausibility is strong and coherent with epidemiology. Vitamin D modulates extravillous trophoblast invasion and spiral-artery remodeling, enhances placental angiogenesis, supports maternal–fetal immune tolerance, and tempers oxidative and inflammatory signaling mechanisms central to normal placentation and nutrient transfer (15–20). Deficiency perturbs these processes and is compatible with the physiologic rise of maternal 1,25(OH)_2_D across gestation that is largely substrate-driven [25(OH)D] (6–9, 15–20). These converging lines of evidence explain why low maternal 25(OH)D is associated with suboptimal fetal growth, even when clinical trials have not consistently shown positive supplementation effects.

Important limitations in the literature temper causal inference. Observational designs, despite multivariable adjustment, remain vulnerable to residual confounding (dietary pattern, adiposity, sunlight exposure, socioeconomic and ethnic factors), single-time-point 25(OH)D measures may miss gestational dynamics, and outcome definitions vary (11–14, 24, 38, 39, 43–52, 58–65). Trials differ in regimen (daily vs. bolus; ∼600–4,000 IU/day), start time, adherence, and background co-nutrients, reducing comparability and diluting pooled effects (4–6, 10–12, 25, 32, 37, 52–57). Overall risk of bias is low-to-moderate across designs and explains a substantial portion of between-study heterogeneity (25–29, 37, 43–51, 53–57).

Progress will depend on assay harmonization and uniform thresholds for 25(OH)D. Inter-laboratory variability, immunoassay vs. LC–MS/MS differences, and inconsistent cut-offs (<20 vs. <30 ng/mL) complicate risk stratification and meta-analytic pooling (11–14, 38, 43–51, 63, 64). We recommend explicit reporting of assay methods and external quality assurance, trimester- and season-specific sampling, and units (ng/mL) with pre-specified deficiency/insufficiency bands aligned to consensus statements to improve comparability and clarify dose–response relationships (22–36).

From a clinical standpoint, risk-based screening is reasonable in populations with high prevalence of deficiency (limited sun exposure, darker skin, higher BMI, restrictive clothing/latitude, dietary insufficiency), coupled with individualized supplementation rather than universal high doses (11–14, 24, 37–39, 52, 58–64). While ∼600 IU/day is commonly recommended, some contexts may require higher intakes (up to ∼4,000 IU/day) to optimize outcomes; however, definitive dose–timing–threshold parameters should be established by harmonized randomized trials with standardized placental and neonatal endpoints and rigorous safety/adherence reporting (10–12, 22–25, 32, 53–57).

Equity and external validity matter. Future studies should prespecify and power subgroup analyses in high-risk groups and expand to low- and middle-income settings where baseline risk, sunlight exposure, and dietary diversity differ, thereby improving generalizability and guiding pragmatic dosing where laboratory monitoring is limited (24–36, 38, 39, 52, 58–64). Co-nutrients (e.g., calcium, magnesium) and gene–nutrient interactions (e.g., VDR polymorphisms) may modify response and merit formal testing via factorial or adaptive designs (19, 20, 23, 37, 56). Methodologic upgrades–dose-ranging/adaptive RCTs, early-pregnancy initiation, standardized 25(OH)D cut-offs and assays, individual-participant-data meta-analyses, and integration of mechanistic placental markers–can accelerate causal inference and translation to practice (23, 25–36, 52–57).

In sum, the weight of evidence supports maternal vitamin D deficiency as a clinically relevant risk marker for impaired fetal growth, underpinned by coherent mechanistic data yet tempered by heterogeneous trial effects. Until precise dose, timing, and 25(OH)D thresholds are defined, targeted screening and individualized supplementation are pragmatic. Harmonized, adequately powered trials with standardized outcomes should resolve remaining uncertainties and inform practice-ready guidance (1–6, 10–18, 21–37, 43–51, 53–57, 65).

Beyond growth outcomes, emerging evidence suggests a pulmonary dimension to maternal vitamin D biology. Animal and translational data link deficiency to impaired fetal lung maturation, particularly reduced surfactant synthesis, raising plausible respiratory implications for preterm or growth-restricted infants that merit targeted clinical evaluation (65). To clarify preventability, forthcoming trials should directly compare early-pregnancy initiation versus continuous supplementation across gestation, incorporate dose-ranging arms, and standardize 25(OH)D assays/cut-offs to define actionable thresholds (20, 23, 27, 52). Regimen choice should favor adherence-supportive daily dosing, with rigorous safety and adherence capture, while testing co-nutrient (e.g., calcium, magnesium) and gene–nutrient (e.g., VDR polymorphisms) interactions that may modify placental and fetal responses (10–12, 19, 20, 23, 37, 53–57). Such harmonized designs–integrating placental vascular/immune/redox endpoints alongside neonatal respiratory outcomes–can move the field from association to practice-ready recommendations.

Most included studies are observational, leaving residual confounding and reverse causation possible despite adjustment. Substantial heterogeneity in assays, cut-offs, sampling timing, and trial regimens (dose, initiation, adherence, co-nutrients) limits comparability and likely contributes to mixed effects. Limited longitudinal measurements and placental endpoints, underrepresentation of diverse settings, and the absence of individual-participant data constrain the assessment of thresholds and effect modifiers. Vitamin D plays an important role in placental angiogenesis, immune modulation, and nutrient transport, which are essential for normal fetal growth and may explain the association between vitamin D deficiency and fetal growth restriction (66).

## 5 Conclusion

In conclusion, this study reinforces the potential link between maternal vitamin D deficiency and FGR, emphasizing the importance of adequate vitamin D levels during pregnancy. While current evidence supports an association, further research is essential to determine causality, refine supplementation guidelines, and establish targeted intervention strategies. A holistic approach integrating maternal nutrition, genetic considerations, and optimal prenatal care is critical for improving fetal growth outcomes and reducing the burden of FGR. Addressing these gaps will not only enhance pregnancy care but also contribute to long-term neonatal health, reducing the risk of chronic conditions associated with intrauterine growth restriction.
